# An RGB colour image steganography scheme using overlapping block-based pixel-value differencing

**DOI:** 10.1098/rsos.161066

**Published:** 2017-04-26

**Authors:** Shiv Prasad, Arup Kumar Pal

**Affiliations:** Computer Science and Engineering, Indian Institute of Technology (ISM), Dhanbad, Jharkhand, India

**Keywords:** colour image steganography, image security, pixel-value differencing, steganography

## Abstract

This paper presents a steganographic scheme based on the RGB colour cover image. The secret message bits are embedded into each colour pixel sequentially by the pixel-value differencing (PVD) technique. PVD basically works on two consecutive non-overlapping components; as a result, the straightforward conventional PVD technique is not applicable to embed the secret message bits into a colour pixel, since a colour pixel consists of three colour components, i.e. red, green and blue. Hence, in the proposed scheme, initially the three colour components are represented into two overlapping blocks like the combination of red and green colour components, while another one is the combination of green and blue colour components, respectively. Later, the PVD technique is employed on each block independently to embed the secret data. The two overlapping blocks are readjusted to attain the modified three colour components. The notion of overlapping blocks has improved the embedding capacity of the cover image. The scheme has been tested on a set of colour images and satisfactory results have been achieved in terms of embedding capacity and upholding the acceptable visual quality of the stego-image.

## 1. Introduction

In the digital world, one of the major and essential issues is to protect the secrecy of confidential data during their transmission over a public channel. In general, the confidential digital data are pre-processed before their transmission over a public channel. This pre-processing operation changes the content of the information into another form, but only an authorized person is capable of appropriately executing the reversible operation on the modified data to retrieve the original content. Several data protection techniques have been devised to protect the confidentiality of digital data. Cryptography [1] is one of the popular techniques used for the secure communication of confidential data. Since the data encryption technique produces a stream of meaningless code for transmission, it may attract an intruder to alter the message intentionally or to retrieve the message by exploiting various cryptographic attacks on the encrypted data.

In contrast, steganography [2] is another mechanism to protect the secrecy of the data. It does not alter the data to make it meaningless to the intruder. In this mechanism, the secret data are embedded into any other unsuspected carrier or cover media like image, audio, video etc. to form a meaningful message that is known as stego-media. It is difficult to distinguish the stego-media from the original cover media by human visual perception. Hence compared with cryptography, the steganographic process prevents an unintended recipient from suspecting that secret data are being transmitted over a public channel through meaningful cover media. A steganography-based security system is used in various applications like military communication, commercial enterprises, Internet of Things and multimedia [3–5]. Several combined cryptographic and steganographic schemes [5,6] are found in the literature. Although the aim of both the cryptographic and steganographic schemes is to ensure data security, the combined approach of cryptography and steganography enhances the security system further with increased computational overhead. So these two security mechanisms, i.e. cryptography and steganography, are exploited distinctly in the field of information security.

Image data are frequently used in various applications. In the literature, a number of image-based steganographic schemes are found to share confidential digital data in a secure way. Among them, the least significant bit (LSB) substitution method [2] is one of the widely used methods due to its simple embedding process and high hiding capacity. In this approach, the least significant bits of the cover pixels are replaced by the secret message sequentially. It has been observed that, up to three bits, LSB [2] replacement is suitable to retain a reasonably good quality stego-image along with high embedding payload. The visual quality of an LSB-based stego-image can be improved further by an optimal pixel adjustment process [2]. Some other LSB substitution-based improved steganographic schemes are found in the literature like a novel scheme proposed by Yang [7]. In that scheme, instead of modifying the cover pixel directly, the secret message bits are inverted and the inverted information known as inverted patterns are recorded for the purpose of extraction of the secret message. Later, Chen [8] suggested an efficient scheme which has improved the visual quality of the stego-image using LSB substitution along with the modulus function approach. In this scheme, the repetition of the secret message is considered for reducing the distortion that occurs in the stego-image. Recently, Xu *et al*. [9] proposed an improved LSB substitution scheme which works on modulo three strategies. The LSB-based steganographic scheme has fixed payload capacity. To improve the payload further, several researchers [10–12] have proposed edge-based steganographic schemes. In natural images, it has been noted that the modification in the smooth region is easily noticeable by human visual perception, and hence hiding more message bits in the edge region is preferred. Such a technique is also proposed by Chen *et al*. [10], where they have developed an edge-based image steganographic scheme where the edge pixels are identified by the combination of the fuzzy edge detector and the canny edge detector, and subsequently, the more secret message bits are embedded in the edge region, rather than the non-edge region, using the LSB method. The combination of the fuzzy edge detector and the canny edge detector has effectively increased the number of edge pixels; as a result, the embedding capacity is high in their proposed scheme. In [11], the authors have distributed image pixels into two categories, i.e. edge pixels and non-edge pixels. A larger number of secret bits are embedded into each edge pixel compared with the non-edge pixels. They have improved the payload capacity but they have compromised with minute visual distortion that occurs in the stego-image. To preserve the high visual quality of the stego-image, the proposed scheme of Islam *et al*. [12] has concealed the secret message bitstreams only at the edge region. In their scheme, the cover image is pre-processed, so that the edge region will be the same even after the embedding of secret message bits. The edge region of the pre-processed cover image is located by a suitable threshold value and that was considered as a stego key. This process enhances the security level further.

Apart from the LSB substitution method, another kind of steganographic scheme was proposed by Wu & Tsai [13] where the secret message was hidden by comparing the differences between the intensity values of two successive pixels. Their method is known as pixel-value differencing (PVD) and it is widely used in the data-hiding field. This method computes the intensity value difference of two consecutive pixels and the hiding capacity is determined based on the pixel value differences. Hence in the PVD technique, more data can be embedded in the edge region in comparison with the smooth region. However, in the smooth region, the hiding capacity is less compared with that of the LSB substitution method. So Khodei & Faez [14] have suggested a combination of LSB and PVD methods where three consecutive pixels are considered in hiding the secret message. Their scheme has improved the embedding capacity and retained the acceptable visual quality of the stego-image. Several other PVD variants [3,15–20] are found in the literature for enhancing the PVD technique. Lee *et al*. [3] have introduced a tri-way PVD approach to improve the hiding capacity and to survive against several steganalyses. Tseng & Leng [15] have modified the traditional PVD-based quantization range table and introduced a new technique known as perfect square number (PSN). The secret message bits are concealed using the PSN and their proposed quantization range table. Liao *et al*. [16] proposed four-pixel differencing and a modified LSB substitution-based steganographic scheme. The edge region pixel is able to tolerate extensively more changes without perceptual misrepresentation than the smooth region. Swain [17] proposed another combination of LSB- and PVD-based improved image steganographic schemes where the secret message bits are hidden into 2 × 2 pixel non-overlapping blocks of a cover image. Recently, another block-based PVD steganographic scheme was presented in [18], where they have considered 3 × 3 non-overlapping image blocks. A seven-directional PVD scheme [19] is found in the literature with improved payload capacity. Conventional PVD suffers from a falling-off boundary problem in some blocks. Hence after the readjustment process, the distortions of those blocks are high when compared with the other blocks. It is of concern that sometimes it provides a low quality of stego-image. Some authors have addressed this problem and their solutions are effective with intensive computational overhead. Zhao *et al*. [20] proposed PVD with modulus function for improving the image quality while preserving the same embedding capacity as found in conventional PVD. Another work is found in [21] where the authors overcome the falling-off boundary problem by adopting the adaptive PVD approach.

Several researchers have employed either LSB substitution or a PVD-based steganographic approach to devise some efficient colour image steganographic schemes. In [22], the authors have enhanced the security of the colour steganographic scheme where they have not concealed secret message bits in sequential order into each colour pixel. The embedding process is realized based on a secret pseudorandom value which decides adaptively the payload capacity and the sequence of embedding secret message bits into each colour plane. Their indirect approach definitely enhances the security level. Another LSB substitution-based colour image steganography is found in [23] where the secret message bits are hidden with reference to an indicator colour plane instead of directly embedding the secret message bits in order. Another secret key-based colour image steganography is suggested by Parvez & Gutub [24] where the secret message bits are spread out over each colour plane based on some predefined secret key. A modified PVD-based steganography is proposed by Nagaraj *et al*. [25]. In their scheme, they used modulus 3 function with PVD for realization of secret message bits into colour pixels. Later, Prema & Manimegalai [26] proposed a colour image steganography using modified PVD. In their scheme, an RGB colour image is decomposed into non-overlapping blocks of two consecutive pixels. Three different pairs, namely (R,G), (G,B) and (B,R), are formed from two consecutive colour pixels and the secret message is embedded based on differences of colour component pairs. They have improved the hiding capacity while maintaining acceptable visual quality of the stego-image. Yang & Wang [27] devised a block-based smart pixel adjustment process where a block of two colour pixels is considered during the secret message-embedding process. However, in their scheme, hiding capacity is not excessive. Adaptive PVD-based colour image steganography is suggested in [28] where the secret message is concealed in the block level of each colour plane. The vertical and horizontal edges are exploited in each block during the message-embedding process. The above colour image steganographic schemes basically work on a colour plane instead of on colour pixels. Hence in this paper, we have proposed an RGB colour image steganography, where the secret message is concealed into each colour pixel independently. The proposed scheme chooses a colour pixel at a time and embeds the secret message into each colour pixel individually by employing the modified PVD appropriately. In the proposed scheme, the colour pixel is grouped into two pairs, namely (R,G) and (G,B), to form two overlapping blocks. PVD is applied to each pair, for embedding the secret message bits. Afterwards, the proposed readjustment process is carried on each pair to obtain the final modified stego colour components, i.e. R, G and B components. The proposed readjustment process ensures that, in the decoding process, PVD is applicable to extract the secret message bits from the stego colour pixel. The proposed scheme will improve the embedding capacity due to consideration of overlapping block concepts.

The rest of the paper is organized as follows. Section 2 presents the basic idea of the PVD method. The details of the proposed scheme are described in §3. The experimental results are presented in §4. Finally, §5 concludes the paper.

## 2. Basics of pixel-value differencing

**Figure 1.**
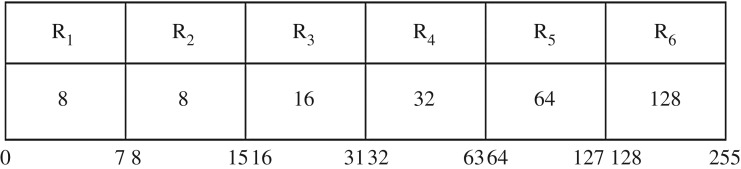
The quantization range.

The PVD method [13] uses grey-level images as the cover image and variable-sized secret message bit sequences are embedded into the cover image. Fewer secret message bit sequences are embedded into the smooth region compared with the edge region. Initially, the cover image is partitioned into non-overlapping blocks of size 1 × 2 in raster scan order. Two consecutive pixels in the *i*th block are denoted as *P_i_* and *P_i_*_+1_, respectively. The difference value, *d_i_*, between two consecutive pixels is calculated by *d_i_* = |*P_i_* − *P_i_*_+1_|. The absolute value of *d_i_* denotes the variation present in each block. A small value of *d_i_* suggests the presence of a smooth region, whereas a larger value indicates the presence of the edge region. The possibility is that *d_i_* belongs to the range of [0, 255] when the greyscale image consists of 256 intensity values. The *d_i_* value can be quantized into several regions as shown in figure 1. The lower and upper bound of each *R_i_* is denoted by [lower*_i_* upper*_i_*]. The number of embedded secret bit sequences (*t*) in two consecutive pixels depends on the quantization range table and it is computed as $t=\lfloor \log_{2}({\mathrm{upper}}_{i}-{\mathrm{lower}}_{i}+1)\rfloor$. The obtained bit sequence is converted into decimal value, *t_d_*. The new difference value $\left(d_{i}^{\prime }\right)$ is obtained by $d_{i}^{\prime }=t_{d}+{\text{lower}}_{i}$.

The modified pixel values are computed based on the following condition:

2.1 $$({P_{i}}^{\prime },P_{i+1}^{\prime })=\left\{\begin{array}{c} \left(P_{i}+\left\lceil \frac{m}{2}\right\rceil ,P_{i+1}-\left\lfloor \frac{m}{2}\right\rfloor \right),\;\text{if}\;P_{i}\geq P_{i+1}\;\text{and}\;d_{i}^{\prime }>d_{i}\\ \left(P_{i}-\left\lfloor \frac{m}{2}\right\rfloor ,P_{i+1}+\left\lceil \frac{m}{2}\right\rceil \right),\;\text{if}\;P_{i}<P_{i+1}\;\text{and}\;d_{i}^{\prime }>d_{i}\\ \left(P_{i}-\left\lceil \frac{m}{2}\right\rceil ,P_{i+1}+\left\lfloor \frac{m}{2}\right\rfloor \right),\;\text{if}\;P_{i}\geq P_{i+1}\;\text{and}\;d_{i}^{\prime }\leq d_{i}\\ \left(P_{i}+\left\lceil \frac{m}{2}\right\rceil ,P_{i+1}-\left\lfloor \frac{m}{2}\right\rfloor \right),\;\text{if}\;P_{i}<P_{I+1}\;\text{and}\;d_{i}^{\prime }\leq d_{i} \end{array}\right\},$$

where $m=|d_{i}^{\prime }-d_{i}|$.

In this method, *i*th block pixels *P_i_* and *P_i_*_+1_ will be replaced by the stego pixels $P_{i}^{\prime }$ and $P_{i+1}^{\prime }$. After the embedding process, the receiver side will compute the difference of the *i*th block $d_{i}^{\prime }=|P_{i}^{\prime }-{P^{\prime }}_{i+1}|$. The difference $d_{i}^{\prime }$ is used to search for the number of concealed bitstreams in the *i*th block using the quantization range from figure 1. The secret bitstreams are obtained after converting the decimal value of ($d_{i}^{\prime }-{\text{lower}}_{i}$) into binary form. An example of the PVD process is illustrated below.

**Example 1.**

**Figure 2.**
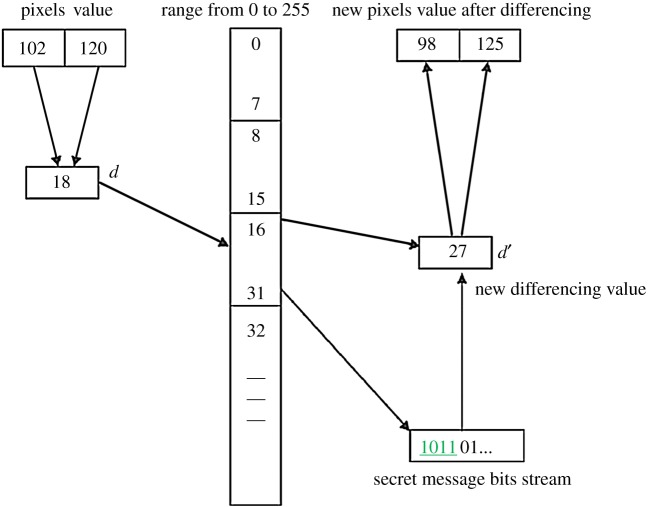
Embedding procedure in the PVD approach.

We illustrate the embedding procedure in figure 2 with a pair of two consecutive pixels 102 and 120 from a cover image. Compute *d* = |120 − 102| = 18 and the lower and upper ranges are searched from figure 1. The difference value, *d* = 18, belongs to the region *R*_3,_ with the corresponding lower = 16 and upper = 31. The number of secret message bits is decided based on

$$t=\lfloor \log_{2}(31-16+1)\rfloor =4\;\text{bits}.$$

Suppose the 4 bits binary secret message is 1011_2_ and its corresponding decimal value is 11_10_. The modified difference and *m* are calculated as follows:

$$\begin{array}{rl} d_{i}^{\prime } & ={\text{lower}}_{i}+\text{Secret message (Decimal)}\\ d_{i}^{\prime } & =16+11=27\\ m & =|d_{i}^{\prime }-d_{i}|=|27-18|=9. \end{array}$$

Finally, as per equation (2.1), the stego pixels will be computed as follows:

$$\begin{array}{rl} \left(P_{i}^{\prime },P_{i+1}^{\prime }\right) & =\{(102-4,120+5),\;\text{if}\;102<120\;\text{and}\;27>18\}\\ \left(P_{i}^{\prime },P_{i+1}^{\prime }\right) & =(98,125). \end{array}$$

The above example is graphically represented in figure 2. In the extraction process, the difference *d*′ = |98–125| = 27 and it belongs to region *R*_3_. The number of embedded secret bits is computed based on the lower and upper value of *R*_3_ where $t=\lfloor \log_{2}(31-16+1)\rfloor =4\;\text{bits}$. The decimal value of the secret message is (*d*′ − lower) = 27 − 16 = 11 and the corresponding 4 bits binary representation is 1011_2_.

## 3. Proposed scheme

**Figure 3.**
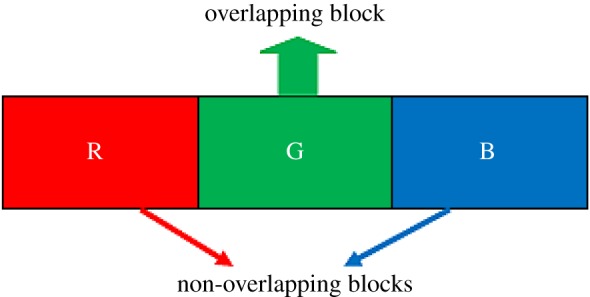
RGB pixels block of colour image.

**Figure 4.**
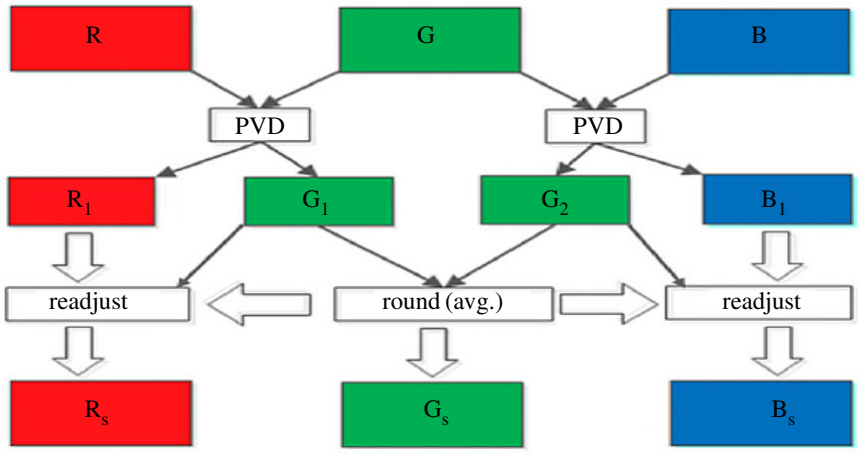
A schematic diagram of data-embedding procedure*.*

**Figure 5.**
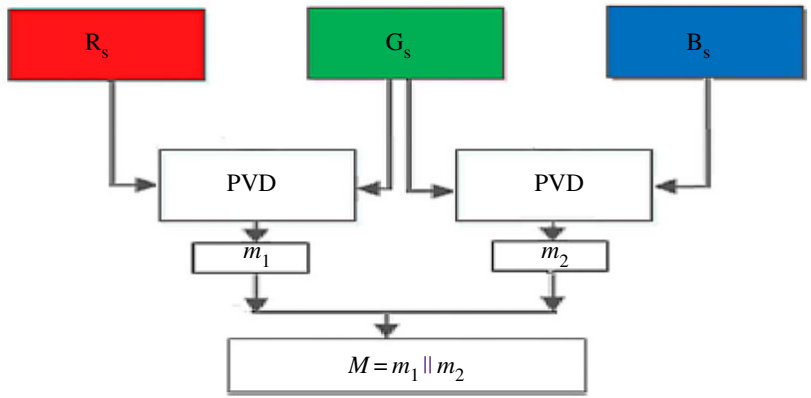
A schematic diagram of data extraction procedure.


The proposed colour image steganographic scheme is presented in this section. Initially, each colour pixel is decomposed into its corresponding colour components, i.e. R, G and B. Later we have formed two pairs with a combination of (R,G) and (G,B). Other ordered pairs are also acceptable, but in this work, we have implemented our scheme using the pairs like (R,G) and (G,B). (R,G) and (G,B) will form two consecutive overlapping blocks as shown in figure 3. In our scheme, we have embedded the variable secret message bits based on the difference of each pair using PVD. After embedding the secret message bits into each pair, the intermediate colour components are further readjusted to attain the final stego-colour components. A natural colour image may be dominated by particular colour components as an outcome of the data hiding process of that particular pixel, and the distortion may be large enough to be perceived. In this paper, we have avoided this circumstance by adopting a suitable threshold value. The data-hiding capacity in each colour pixel is restricted by the threshold value, so that the stego-image may retain high visual quality. Figure 4 shows the overall embedding process. The decoding process is shown in figure 5. The algorithm steps of the proposed embedding and extraction procedure are presented as follows:

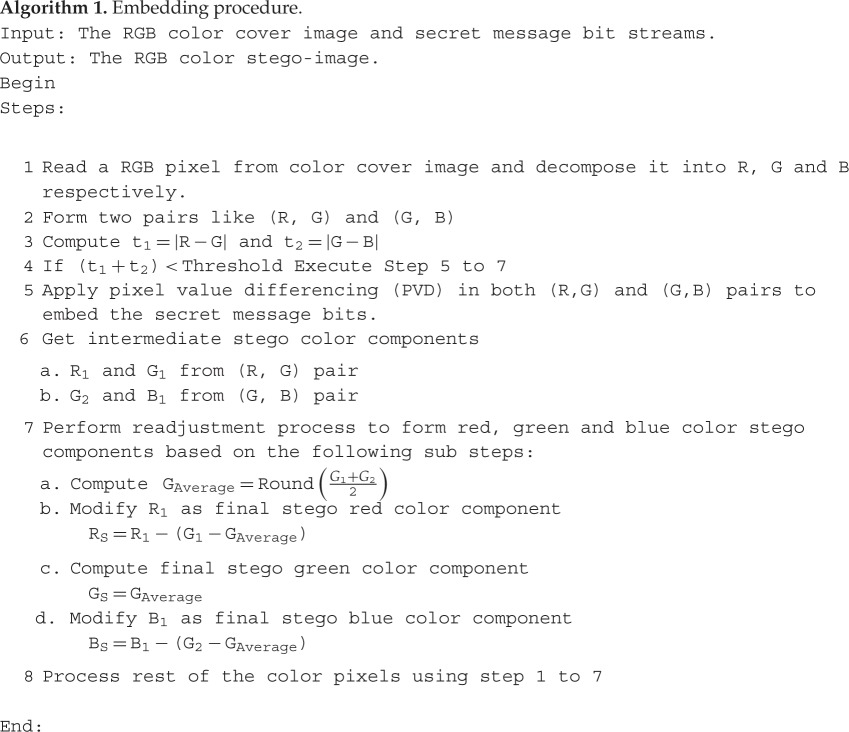

**Example 2**

**Figure 6.**
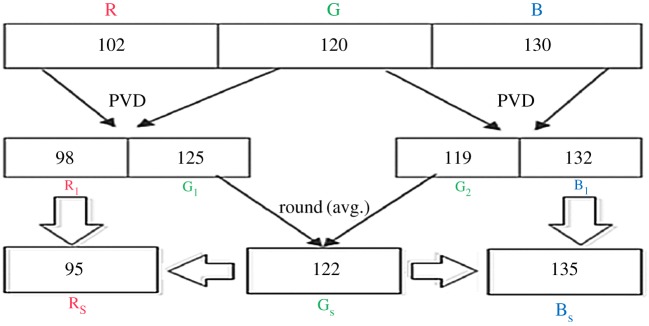
Example of the embedding procedure.

An illustration of the secret message-embedding procedure is given in figure 6. Let the RGB colour components be 102, 120 and 130, respectively. We have taken a random secret message bitstream as 10111011100111…. The (R,G) pair has embedded the secret bitstream as 1011_2_ based on the PVD approach. The other pair (G,B) has selected the secret message bits as 101_2_ based on the PVD approach. The stego colour components obtained according to our approach are 95, 122 and 135.

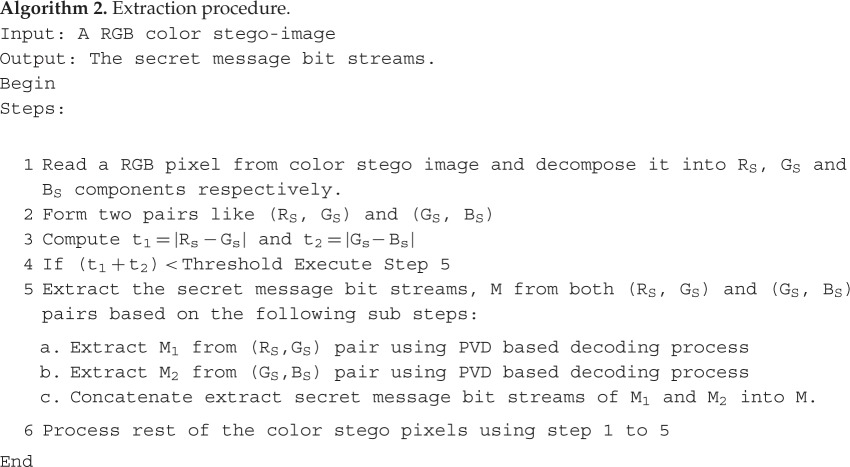


## 4. Experiment results

**Figure 7.**
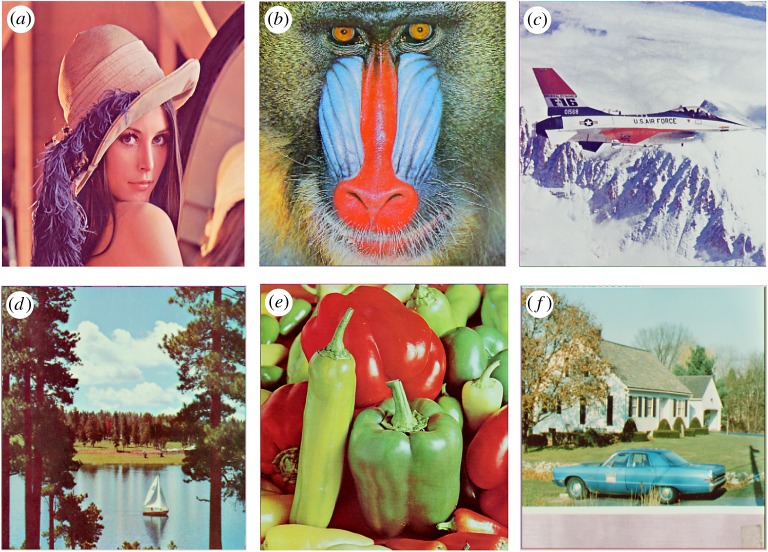
Original cover images used in the experiment.

**Figure 8.**
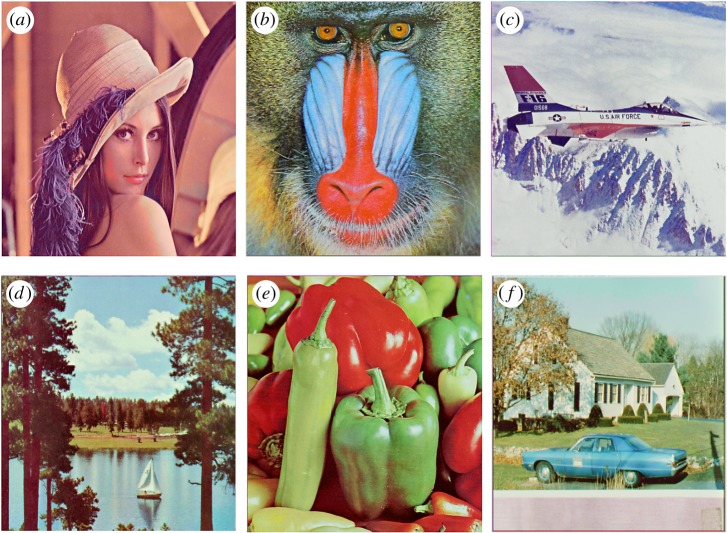
Stego-images after data hiding.

**Figure 9.**
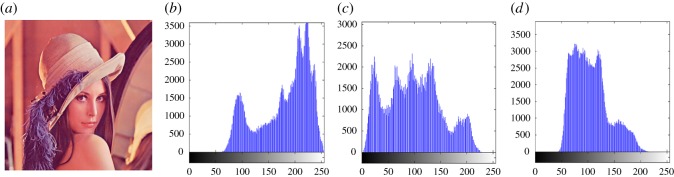
(*a*) Lena cover image. (*b*–*d*) Histograms of red, green and blue components.

**Figure 10.**
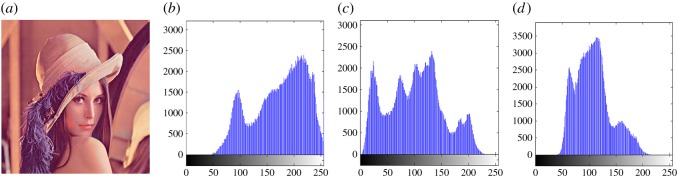
(*a*) Lena stego-image. (*b–d*) Histograms of red, green and blue components.

**Figure 11.**
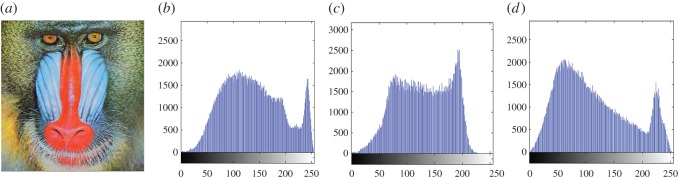
(*a*) Baboon cover image. (*b*–*d*) Histograms of red, green and blue components.

**Figure 12.**
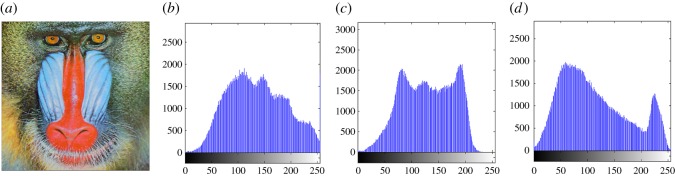
(*a*) Baboon stego-image. (*b–d*) Histograms of red, green and blue components.

**Figure 13.**
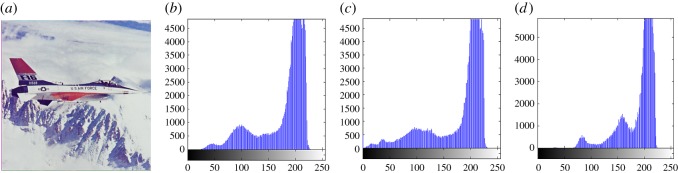
(*a*) Jet cover image. (*b*–*d*) Histograms of red, green and blue components.

**Figure 14.**
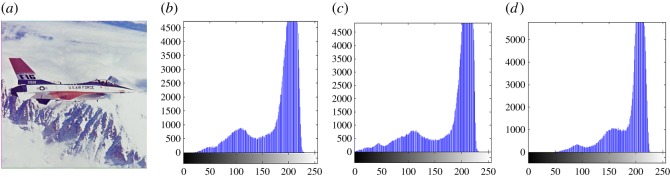
(*a*) Jet stego-image. (*b*–*d*) Histograms of red, green and blue components.

**Figure 15.**
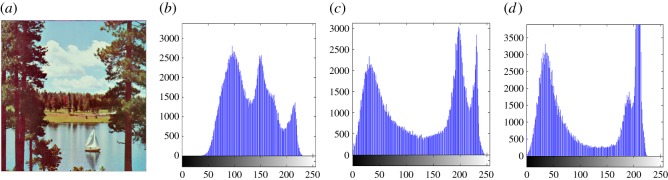
(*a*) Sailboat cover image. (*b*–*d*) Histograms of red, green and blue components.

**Figure 16.**
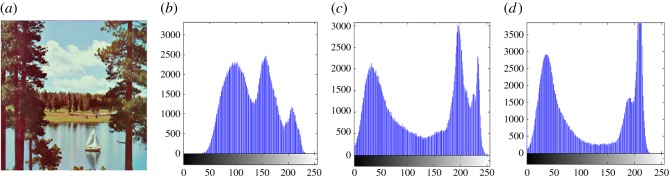
(*a*) Sailboat stego-image. (*b*–*d*) Histograms of red, green and blue components.

**Figure 17.**
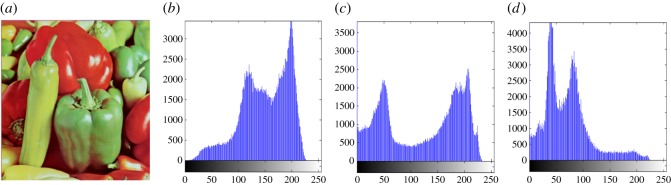
(*a*) Pepper cover image. (*b*–*d*) Histograms of red, green and blue components.

**Figure 18.**
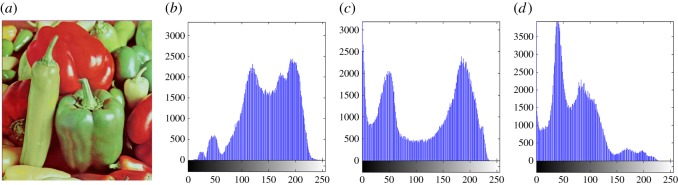
(*a*) Pepper stego-image. (*b*–*d*) Histograms of red, green and blue components.

**Figure 19.**
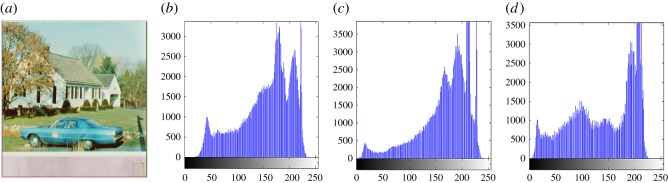
(*a*) Car–house cover image. (*b*–*d*) Histograms of red, green and blue components.

**Figure 20.**
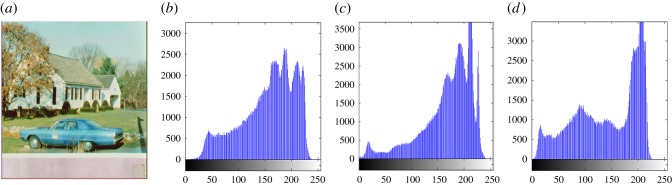
(*a*) Car–house stego-image. (*b*–*d*) Histograms of red, green and blue components.

**Figure 21.**
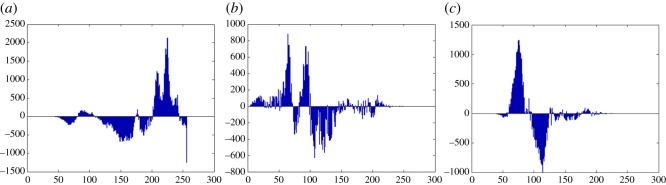
Lena difference image histograms are (*a*) R, (*b*) G and (*c*) B.

**Figure 22.**
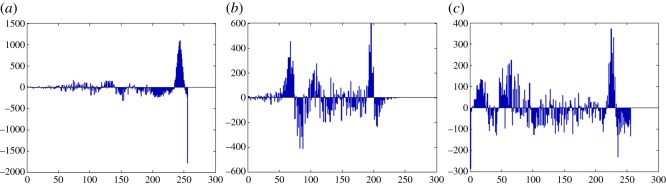
Baboon difference image histograms are (*a*) R, (*b*) G and (*c*) B.

**Figure 23.**
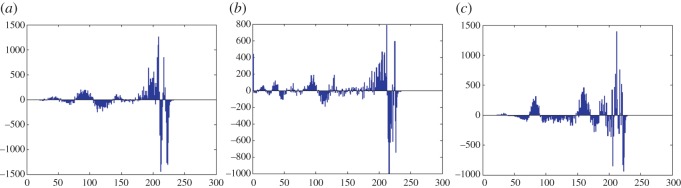
Jet difference image histograms are (*a*) R, (*b*) G and (*c*) B.

**Figure 24.**
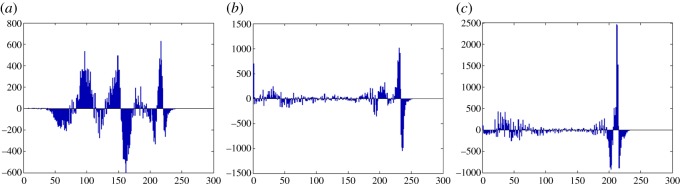
Sailboat difference image histograms are (*a*) R, (*b*) G and (*c*) B.

**Figure 25.**
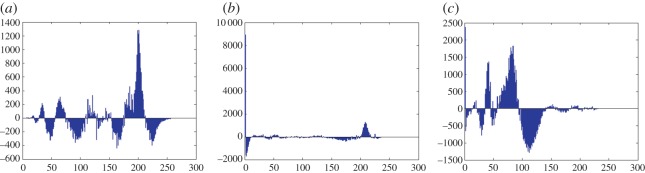
Pepper difference image histograms are (*a*) R, (*b*) G and (*c*) B.

**Figure 26.**
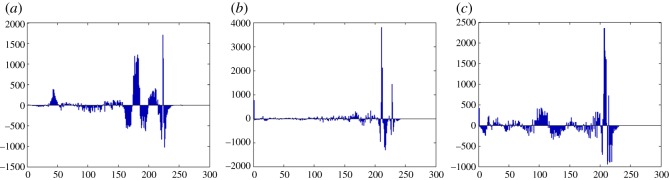
Car–house difference image histograms are (*a*) R, (*b*) G and (*c*) B.

In this section, the experimental results are presented to demonstrate the performance of the proposed scheme. The proposed scheme has been tested on a set of standard colour images, but in this paper, we present the results for six colour images where the images are selected with consideration of diverse image features to estimate the performance in terms of visual quality and embedding capability of the stego-images. The original images are shown in figure 7. The randomly generated message bits are considered as secret message bitstreams in our experiment. After the embedding process, the obtained stego-images are as shown in figure 8 and it is observed that the imperceptibility of stego-images is high. The histograms of the original cover image and stego-images are depicted in figures 9–20 and the plotted histograms reveal similarity between original and stego-images. Figures 9–20 suggest that, in our proposed scheme, the disparities occurring due to embedding of secret message bitstreams are not noticeable in the stego-image. In addition, the differences occurring in histogram levels are reasonably insignificant, as shown in figures 21–26. The stego-image quality is further estimated in terms of the peak signal-to-noise ratio (PSNR) and embedding capacity/payload. Table 1 gives the results of the proposed scheme in terms of embedding capacity and PSNR value. We have obtained high acceptable PSNR values for stego-images with a high embedding capacity of secret messages. Hence, in the proposed scheme, the PSNR values as well as the visual appearance of the stego-image and histogram suggest that the distortion appearing after embedding of the secret message into the cover image is reasonably less and imperceptible to human visual perception. The proposed scheme is also compared with some other steganographic schemes in terms of embedding capacity and PSNR, and their results are given in table 1. The experimental results indicate that the proposed steganographic scheme appropriately meets the requirements of steganography, where we have succeeded to embed a huge number of secret bitstreams while maintaining acceptable visual quality of stego-images.

**Table 1.** The simulation results.

	PVD method [13]		Yang & Wang [27]		Mandal & Das [21]		Swain's proposed method 1 [28]		proposed method	
cover image (512 × 512 × 3)	capacity (bits)	PSNR (dB)	capacity (bits)	PSNR (dB)	capacity (bits)	PSNR (dB)	capacity (bits)	PSNR (dB)	capacity (bits)	PSNR (dB)
Lena	1 234 394	41.25	196 608	41.58	1 234 394	40.21	1 341 192	46.17	1 976 671	31.01
baboon	1 406 405	37.81	196 608	33.29	1 406 405	37.14	1 489 945	48.49	2 219 715	32.29
jet	1 224 178	40.44	196 608	43.73	1 224 178	40.64	1 267 690	46.18	1 753 707	35.66
sailboat	1 289 871	38.76	196 608	47.41	1 289 871	39.35	1 424 967	47.29	2 130 772	33.11
peppers	1 236 715	40.31	196 608	39.43	1 236 715	40.37	1 350 251	47.06	1 783 210	30.10
car–house	1 263 038	38.97	196 608	41.34	1 263 038	39.62	1 339 985	44.73	2 079 088	34.59
average	1 275 766	39.59	196 608	41.13	1 275 766	39.55	1 369 005	46.65	1 990 527	32.79

## 5. Conclusion

Most colour image steganography works on individual colour components instead of considering all colour components together. But in this paper, the proposed method conceals the secret message bits directly into each pixel sequentially. Conventional PVD works on the idea of overlapping blocks of colour components. The proposed readjustment process of colour components confirms the feasibility of conventional PVD-based decoding procedure. The experimental results reveal that the proposed scheme has a larger hiding capacity with acceptable imperceptibility of the stego-image. In addition, the proposed scheme is simple and easy to implement on RGB colour images.

## Data accessibility

Our data have been deposited at Dryad (http://dx.doi.org/10.5061/dryad.21tm5) [29].
